# Ethics support personnel’s perceptions of patient and parent participation in clinical ethics support services in pediatric oncology

**DOI:** 10.1186/s12910-025-01267-5

**Published:** 2025-07-19

**Authors:** Isabelle Billstein, Cecilia Bartholdson, Anders Castor, Bert Molewijk, Pernilla Pergert

**Affiliations:** 1 Department of Women’s and Children’s Health, Childhood Cancer Research Unit, Karolinska Institutet, Tomtebodavägen 18A, Stockholm, 171 77 Sweden; 2 Astrid Lindgren Children´s Hospital, Karolinska University Hospital, Stockholm, Sweden; 3 Childhood Cancer Center, Skane University Hospital, Lund, Sweden; 4 Department of Ethics, Law & Humanities, Amsterdam University Medical Centers & VU University, Amsterdam, the Netherlands; 5 Centre for Research Ethics & Bioethics (CRB), Department of Public Health and Caring Sciences, Uppsala University, Uppsala, Sweden

**Keywords:** Pediatrics, Ethics, clinical, Patient participation, Parents, Psycho-oncology, Qualitative research

## Abstract

**Background:**

There is an ongoing discourse about patient and parent participation (PPP) in Clinical Ethics Support Services (CESS), and this paper focuses specifically on case-based CESS. Participation in CESS is increasing slowly in many contexts due to practical and moral complexity. To gain deeper understanding of PPP in CESS, we need to delve into stakeholders’ perspectives and the landscape in which they operate. The aim of the study was to explore perceptions regarding feasibility and moral appropriateness of PPP in CESS in pediatric oncology.

**Methods:**

Nordic healthcare personnel (*n* = 26) working as ethics support personnel in pediatric oncology (and/or pediatrics in general) participated in focus group interviews (*n* = 6). Data was analyzed with qualitative inductive content analysis.

**Results:**

Despite engagement in CESS, most ethics support personnel had no former experience of PPP in CESS. The ethics support personnel expressed potential benefits with PPP in CESS, but these were overshadowed by fear of causing participant harm. The potential benefits and harms included to deepen understanding and trust, to catalyze confrontation and to create dilemmas of decision-making participation. Reported strategies to mitigate potential negative consequences and reduce risk of causing harm were at organizational, relational and individual levels.

**Conclusions:**

Despite seeing positive reasons for PPP in CESS, the ethics support personnel were mainly concerned about the potential participant harm and wanted to protect the child and the parent. This could be interpreted as a form of disguised paternalism. The perceived appropriateness of PPP in CESS in pediatric oncology seems to depend on the situation. Furthermore, in cases where it can be considered, there is no universal way of doing it. An important enabler may be to customize PPP in CESS on a case-by-case basis and to apply the identified strategies to reduce potential risk of causing harm. This study contributes to increased knowledge about PPP in CESS from the perspectives of ethics support personnel in pediatric oncology and informs us about what is needed to carefully foster PPP in CESS, both practically and morally.

**Supplementary Information:**

The online version contains supplementary material available at 10.1186/s12910-025-01267-5.

## Background

The aim of Clinical Ethics Support Services (CESS) is to assist in the management of ethically difficult situations [1]. Although there are different procedures for CESS, its cornerstones consist of identifying, examining and resolving ethical dilemmas [2]. CESS can be offered by clinical ethics committees [3], clinical ethics consultants [4] or through facilitated reflections like moral case deliberations [5], also referred to as ethics case reflections [6]. Clinical ethics committees often work at an organizational and/or policy level, but can also educate in ethics or be consulted in patient cases [7]. However, henceforth, this paper will focus on patient case-based CESS, thus not CESS activities on organizational level. Clinical ethics consultants perform ethics consultations on an individual level and are often bioethicists specialized in clinical practice or clinicians specially trained in bioethics [8]. Ethics consultations involve a rather top-down process including an individual expert approach. Whereas facilitated group reflections can be seen as a bottom-up process, focusing specifically on the moral learning and moral competence of participants while reflecting upon a specific case and its moral question [5, 9]. In facilitated reflections, the conversation usually follows a chosen structured dialogue method, preferably led by a certified facilitator [5, 9]. Since many ethical issues and dilemmas concern patients and their moral viewpoints about quality of care and decision-making, questions of patient participation in CESS need to be raised.

To deliberate on patient participation in CESS, we need to begin with elaborating on the ambiguous concept of participation in healthcare. Participation can be defined as “patient’s rights and opportunities to influence and engage in the decision making about his care through a dialogue attuned to his preferences, potential and a combination of his experiential and the professional’s expert knowledge” [10, p. 1929]. Increased participation in healthcare can have several positive effects; for instance, it can enable patients to exercise their autonomy, improve the care-relationship and clarify the patients’ experiences and expectations of care [11]. From a pediatric perspective, Hart’s “Ladder of Children’s Participation” [12] is a well-known theory of children’s participation. Shier [13] further developed this model by describing five levels of pathways to participation, ranging from listening to children to shared power/responsibility for the decision-making. The practice and level of participation may depend on both organizational and individual conditions. For example, it was shown in a pediatric interview study that physicians and parents were the ones deciding on the level of children’s participation in decision-making situations [14]. Moreover, increased participation has also been interrelated with protection and promotion of the child’s health [15].

During the last decades, there has been an ongoing discourse and growing interest in patient and family participation in CESS in adult as well as pediatric care [16–20]. Since an ethics analysis in CESS involves clarification of all relevant facts and perspectives in the case [5, 9], something that healthcare personnel alone might not be able to provide, one can argue that patient and family participation in CESS is needed from both a theoretical and practical perspective. As ethical dilemmas can be experienced by children [21] and family members [22, 23], there are also moral arguments that support patient and family participation in CESS. Additionally, healthcare personnel have ranked children and family members as the second most important participant in CESS [24], which also supports increasing participation. Thus, without proper space for patient and family participation in CESS, there is a risk of overlooking essential perspectives including important ingredients for the determination of quality of care. However, there is a controversy of how and when participation in CESS ought to be performed and which kind/method of CESS to use. Already known challenges of transparency of CESS between healthcare personnel and patients involves, for example, questions of how to document healthcare personnel’s sensitive information and perceptions of the patient in the medical record [25]. Further, pitfalls like the patient’s perspectives and treatment preferences dominating the moral deliberation and the risk of revealing confidential information to participants not involved in the patient’s care, have also been found causing worries [26]. This, together with a concern that patients and family members might experience a burdensome responsibility for tough decisions [26], reinforces further exploration of patient and family participation in CESS.

Patient and family participation in CESS varies depending on the context and procedure of CESS [26]. Considering ethics support by ethics consultants and committees, participation in terms of meeting with the patient and/or family members or inviting them into the ethics meeting seems to be well developed in North America [27], and some European countries like Norway [20, 28], Germany and France [26]. Although having practices and routines for this kind of patient and family participation in CESS, research has shown that patients and families rarely request it [27] which creates questions of awareness and accessibility that might be limiting participation. Moreover, in other CESS contexts, patient and family participation seems challenging [16, 26] indicating room for improvement. Considering facilitated reflections, it seems difficult to find studies examining the role of patients and families. However, in a recent Dutch study [17], which is a context where moral case deliberations have been frequently used, the level of patient participation was quite low. Further, in pediatric settings, it seems difficult to find studies that examine patient and family participation (which henceforth will be referred to as patient and parent participation (PPP)) in CESS.

In Sweden, there has been an on-going project (predominantly led by some of the authors) to implement and develop CESS in pediatric oncology [9, 29, 30], as well as to explore dilemmas of involved stakeholders [21, 23]. In Swedish pediatric oncology there can be different procedures and routines for CESS including ethics committees, ethics groups and teams. In 2017, however, the Nordic Society of Paediatric Haematology and Oncology’s working group on ethics introduced moral case deliberations to all six pediatric oncology centers. This was done by inviting healthcare personnel to a joint education/training which included two gatherings of three and two days of training, together with a period of practice at the trainee’s home center between the gatherings. Besides doing the facilitating of moral case deliberations, healthcare personnel were also responsible for the implementation of moral case deliberation [31]. Since then, the moral case deliberation facilitator training has been offered repeatedly. Up to this point, however, participation in CESS offered in Swedish pediatric oncology has been limited to healthcare professionals.

## Aim

The aim of the study was to explore perceptions of ethics support personnel (ESP) regarding the feasibility and moral appropriateness of PPP in CESS in pediatric oncology. The following research questions were explored: (1) What are ESP’s perspectives and attitudes towards PPP in CESS, and which promoting and inhibiting factors do they mention? (2) How can PPP in CESS be organized according to ESP?

## Methods

### Study design and setting

This empirical, explorative, qualitative and descriptive study was conducted in the context of Nordic, and predominantly Swedish, pediatric oncology.

### Study participants

A total of 33 ESP with experience of providing CESS in Nordic pediatric oncology (and/or pediatrics in general), were invited to focus group interviews. In this paper ESP were defined as personnel educated and/or experienced in CESS. The ESP were invited due to their knowledge of CESS and their role as key persons in a potential PPP implementation. All participating ESP (*n* = 26) answered the sociodemographic questionnaire (Table 1).

**Table 1:** Characteristics of the ESP (*n* = 26)

Characteristics		*n*		(%)^*^
Sex (*n* = 26)	Woman		20	(77)
	Man		6	(23)
Profession (*n* = 25)	Reg. nurse		11	(44)
	Physician		11	(44)
	Other		3	(12)
CESS education (*n* = 26)	Yes/ongoing		23	(89)
	No		3	(12)
Years of CESS experience (*n* = 26)	< 1		7	(27)
	1–5		16	(62)
	6–10		2	(8)
	> 10		1	(4)
Hours/week performing CESS (*n* = 26)	< 1		18	(69)
	1–5		6	(23)
	6–10		2	(8)
Patient care in their assignment (*n* = 26)	Yes		25	(96)
	No		1	(4)

^*^ Total percentage may be > 100% due to rounding errors

The ESP included facilitators of moral case deliberations (*n* = 13 in 3 groups) who worked at or in connection with the six Swedish pediatric oncology centers, all of whom participated in a facilitator workshop. Further, members of two Swedish pediatric ethics committees (*n* = 12) were invited of which five were interviewed in two separate groups during one of their regular meetings. Lastly, eight members that participated in a regular meeting of the Working Group on Ethics from pediatric oncology, representing all Nordic countries, were invited of which all participated. The ESP’s most common role in CESS were as facilitators of moral case deliberations, ethics representatives and members of ethics groups/committees at local, national and international level. About half of the ESP (*n* = 14) worked with CESS in plural roles.

### Data collection and analysis

The study was approved by the Swedish Ethical Review Authority (No: 2022-06171-01). Data was collected between January and November 2023, using focus group interviews (*N* = 6, 2–8 participants/group) which were performed in-person (4 groups) or digitally (2 groups). Focus group interviews were planned to consist of a minimum of three participants, but due to last-minute withdrawal one focus group only had two participants. Focus group interviews were divided by center or working group to facilitate data collection and stimulate reflection of one’s own clinical setting. In the facilitator workshop, facilitators from different centers were grouped to create appropriately sized groups. Written and oral information about voluntary participation and confidentiality was provided. Written consent was collected, and all participants consented to audio recording of the interviews. Interviews lasted between 64 and 122 min and were preceded by a short introduction to the research project. Interviews were conducted by all authors and facilitated using dual moderation, with one researcher primarily facilitating the discussion, and the other assisting with descriptive field notes [32]. To structure the interviews, the research team had created an interview guide [see Additional file 1] with questions about PPP in CESS in general and fictitious patient cases where ethical dilemmas between healthcare personnel, parents and children were discussed.

The analysis consisted of three phases: preparation, organization, and reporting. In the preparation phase, interviews were transcribed verbatim (by the first author) and de-identified at person level, and together with field notes imported into a software for qualitative analysis (NVivo version 13). As the research field of PPP in CESS in pediatric oncology is relatively unexplored, data was analyzed using inductive content analysis, starting the organization phase with open coding [33]. This analysis method was chosen as it generates knowledge that serves to be applicable in its context [34]. Thereafter, codes were grouped into sub-categories and sub-categories into generic categories, and a main category. Each step included a higher level of abstraction on what was going on in the data. Throughout the process, memos were written and the memos about the sub-categories and generic categories formed the initial version of the results. Validation was performed in each step by all authors. Validation of the final result was also performed together with the first, second and last author’s research group colleagues, who were all acquainted with the method or the research area.

## Results

Most ESP had no previous experience of PPP in CESS; their perceptions were based on clinical experiences in working with patients and parents as healthcare personnel, and as ESP in pediatric oncology (and/or pediatrics in general). An overview of the categories can be found in Table 2.

**Table 2:** Overview of categories

Main category	Generic categories	Sub-categories
Potential benefits were overshadowed by fear of causing harm	Potential benefits and harms	Deepen understanding and trust
		Catalyze confrontation
		Create dilemmas of decision-making participation
	Strategies to avoid causing harm	Create organizational conditions
		* Challenge the ambiguity of ethics and CESS*
		* Prepare and debrief*
		* Create a safe environment*
		Handle relational roles and input values
		* Balance care relationship*
		* Protect child-parent relationship*
		Protect integrity
		* Assess child participation*
		* Assess parent participation*
		* Assess exposure of healthcare personnel*

### Potential benefits were overshadowed by fear of causing harm

In this study, the ESP’s overall attitudes of PPP in CESS were that the potential benefits were overshadowed by fear of causing participant harm. For most ESP, PPP in CESS in pediatric oncology was considered controversial and led to intensive discussions. The ESP’s perceptions expressed were in terms of potential benefits and harms, of which the harmful consequences outweighed the beneficial ones and influenced the interviews to a large extent. The ESP suggested strategies on different levels (organizational, relational and individual) to reduce the potential risk of causing participant harm.

### Potential benefits and harms

This generic category consists of three sub-categories with sub-headings, and describes perceived potential benefits and harms of PPP in CESS; the ESP stated that PPP in CESS could deepen understanding and trust, catalyze confrontation and create dilemmas of decision-making participation. They perceived PPP in CESS to be positive in theory, however, it was perceived complex to perform in practice.

To ***deepen understanding and trust*** between participants was seen as a potential consequence of PPP in CESS. For the ESP, PPP could give the family a chance to formulate and express their values and norms. In CESS with PPP, the family would be considered as the best representative of their unique situation, and their firsthand information could clarify values at stake and deepen the understanding of the situation, especially for the decision-maker. It was expressed that PPP in CESS could reduce the risk of misinterpretation and/or over-interpretation of families’ values and norms, something that the ESP sometimes experienced in CESS without PPP. One participant said: *“…because we [the healthcare personnel] also… overinterpret ‘this is what the parents think*,* this is what the child probably thinks’ and we have no idea” (focus group interview (FGI) 2).* The deepened understanding could lead to an increased feeling of trust and safety which could provide families with a better experience of healthcare. However, if families during CESS gained deeper understanding of the limitations of healthcare and the healthcare personnel’s doubts and concerns, this could also create negative consequences.

To ***catalyze confrontation*** was another perceived potential consequence of PPP in CESS, and the ESP deliberated that this confrontation could mitigate as well as exacerbate potential conflicts between participants. The ESP thought that potential conflicts could be mitigated and easier to handle due to the deepened understanding and acceptance of differences and decisions. However, the confrontation between participants in PPP in CESS was also seen as a potential source of conflicts, especially if the dilemma involved differences in cultural aspects or principles like cost effectiveness. One participant described that: *“…it can be very frustrating*,* especially if there also are cultural differences*,* because then it can be so difficult to achieve any kind of consensus or common ground” (FGI 1).*

The likelihood to ***create dilemmas of decision-making participation*** was a frequently expressed potential consequence of PPP in CESS, as the ESP perceived that CESS serves to assist in decision-making processes. On the one hand, PPP in CESS could be seen as an ethical value of its own as children and parents’ ought to have the right to discuss and understand on what grounds a decision is made. PPP in CESS was also perceived to lead to a broader/more correct decision-making basis for the decision-maker. On the other hand, shared decision-making, or even insight into the decision-making process involved in CESS, could be harmful for families: *“…we [the healthcare personnel] have to take some responsibility of shielding patients and parents from the information that we sincerely*,* with all moral and emotional being*,* we really sincerely think that is not good for you to know” (FGI 3).* It was considered unacceptable to put a family in a situation where they could feel guilty for having contributed to a decision (which was assisted by PPP in CESS) that caused the child harm. Regarding some ethical challenges (for example cost-effectiveness), the ESP thought that PPP in CESS should not be considered at all since they thought that the decision should solely be made by healthcare personnel.

### Strategies to avoid causing harm

**Fig. 1:**
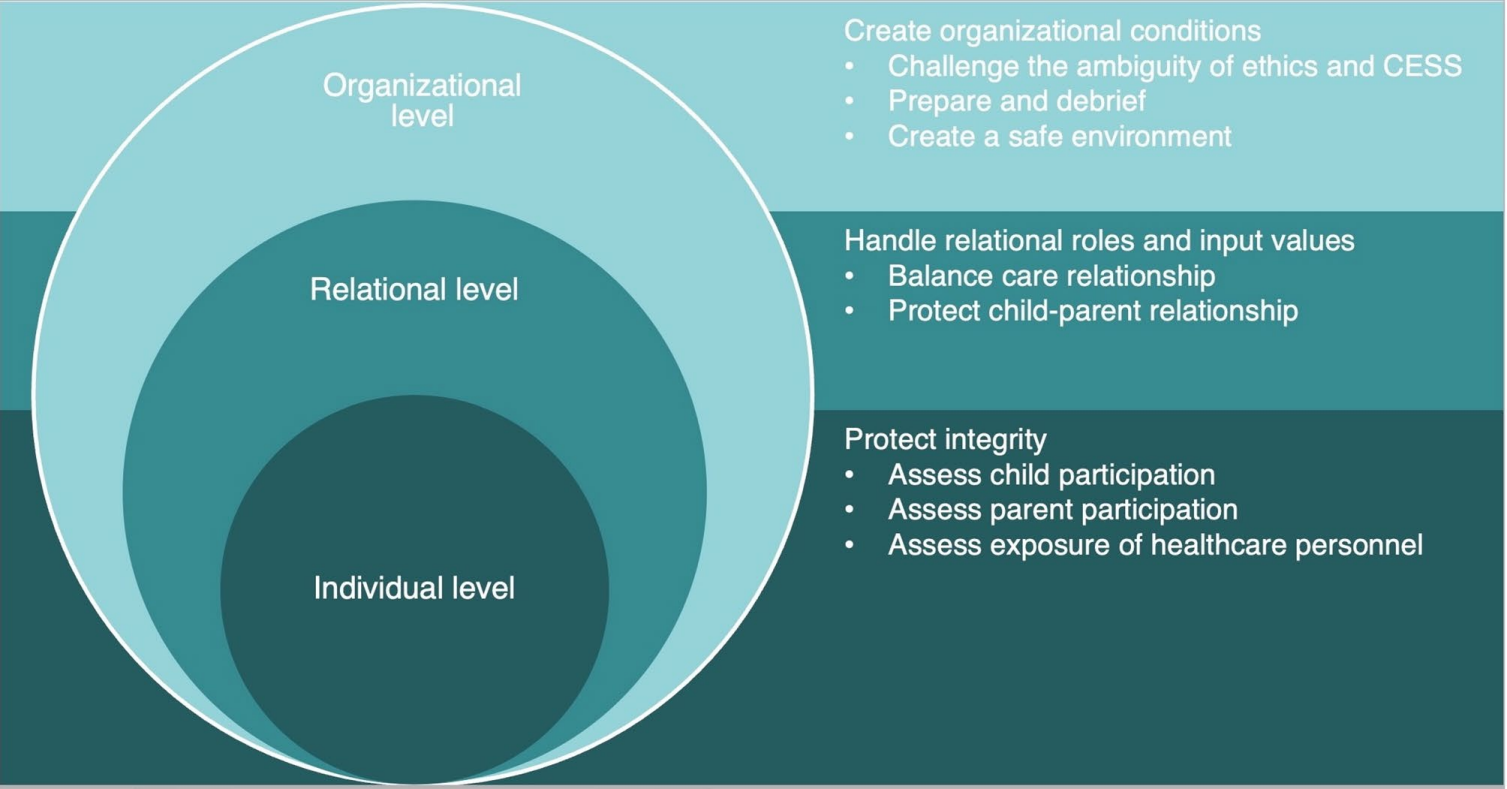
Model of the ESP’s perceived strategies to reduce the risk of causing harm expressed on different levels

This generic category originated from three sub-categories and describes strategies of PPP in CESS to mitigate potential participant harm described at the organizational, relational and individual level (Fig. 1). The model of perceived strategies is inspired by Bronfenbrenner’s ecological model which shows how individuals are inter-related by organizational and relational conditions [35, p. 3–15].

The sub-category to ***create organizational conditions*** describes perceived existing conditions and conditions needed to be created to enable PPP in CESS.

To *challenge the ambiguity of ethics and CESS* was a strategy that the ESP thought could be beneficial to enhance the introduction of PPP in CESS. The ESP expressed that among healthcare personnel and families, the concept of ethics was loaded and needed to be dedramatized. One participant described this like: *“The word ethics is also a complicated word and can be a bit pretentious.” (FGI 2).* It was considered difficult to identify ethical dilemmas and their values, something that affected the request for CESS in general, thus also PPP in CESS. This was further highlighted as the ESP had difficulties accepting the ethical dilemmas of family members as genuine ethical dilemmas and, for example, thought they were rather emotional problems and thus suggested couples’ therapy instead of CESS. Additionally, ESP expressed that healthcare personnel sometimes experienced CESS as difficult to understand and perform, which hindered CESS in general but also PPP specifically. Hence, it was considered helpful to start with addressing the ethics in daily care and increase information about CESS. The ESP expressed hesitation to involve families in CESS as they first wanted more confidence and experience in facilitating CESS with healthcare personnel alone.

The ESP suggested that to *prepare and debrief* could be helpful to reduce the risk of causing participant harm. This included choice of CESS activity and method for ethical analysis, which was suggested to be adapted to the unique clinical setting and case. Another perceived enabler was to have an already established trustful and well-functioning care relationship, and that all participants should be well prepared and aware of the situation entering CESS. One participant expressed that: *healthcare personnel could “…tell them [the child/parent] we could have this kind of session where we would use this kind of method and talk about like… to aim for solution and not for disagreements” (FGI 4).* It was underlined that family members should have the opportunity to talk about their experiences afterwards.

To *create a safe environment* when performing PPP in CESS was stated as important. Safety involved mainly the composition of participants and the way of communicating during CESS. For example, low number of relevant participants, together with a neutral and well-experienced facilitator were seen as key factors. One participant said: *“…one of the nice things with the Dilemma method is that you do not sit there as different teams*,* the care team [and] the parent team… we sit as individuals with different perspectives” (FGI 6).* It was considered important to adapt vocabulary and content of discussion based on the child’s or parent’s ability and interests. However, adaptation of content discussion was also seen as a risk of impairing the ethics reflection.

The sub-category to ***handle relational roles and input values*** derived from the ESP’s perception that stakeholders’ differences affect interactions which must be considered when performing PPP in CESS.

To *balance care relationship* appeared to be important for the ESP, and any risk of jeopardizing the care relationship worried many of them. To enhance participant interactions, it was considered important to alert hierarchal structures and stakeholders’ relational roles and differences in input values – this as it could affect how perspectives, knowledge and values are expressed and respected among participants. One participant described that *“It is about their lives*,* for us it is our job. We can get another job tomorrow.” (FGI 1).* It was highlighted that all adult participants have responsibility for maintaining the care relationship.

To *protect child-parent relationship* was considered important to reduce risk of causing harm. The ESP described the initial family constellation and relationship as important factors, but also how the relationship had been affected by the cancer diagnosis and the course of care. The protection and dependency between children and parents advocated to offer both a forum of their own where they could speak freely without concerns of each other: *“The child wants to protect his parents and does not want to say what he is thinking and so on*,* so it is very much that they [the child and the parent] protect each other in some way more or less.” (FGI 5).* However, the ESP’s views varied, and some underlined the importance of sharing perspectives together.

The sub-category ***protect integrity*** encompassed the ESP’s descriptions of efforts to safeguard the integrity and interests of stakeholders.

To *assess child participation* before PPP in CESS was frequently expressed to protect the integrity of the child. This included his/her ability to participate in CESS with respect to age and maturity, as well as the appropriateness of the ethical dilemma. This assessment was suggested to be assisted by the parent/s: *“If it is a slightly older child*,* you should probably ask the child*,* but you should perhaps also ask the parent for their opinion*,* because they know their child best after all.” (FGI 5).* Some ESP proposed that children, in contrary to other CESS participants, should have the opportunity to only state their perspectives and not be forced to hear adults’ ones.

The ESP also suggested to *assess parent participation*, and this was a question of the appropriateness of the ethical dilemma as well as the parent’s cognitive and psychosocial prerequisites to handle the CESS activity. It was important to, in advance, explore what kind of information the parent wants to take part in since it could affect the organization of PPP in CESS. One participant emphasized that: *“There is such a wide range of parents.” (FGI 6).* The ESP perceived that an important goal of PPP in CESS is to improve the ethics analysis, and that this could be done by assisting the parents to reflect and formulate their ethical values. Thus, it was perceived that their attendance should be of importance for the dialogue and other participants.

The perceived importance to *assess exposure of healthcare personnel* involved managing healthcare personnel’s feelings of exposure towards the families regarding their own ethical values and thoughts. One participant expressed: *“They [patients/parents] have the power to leave at any time… I have to do this as a part of my job. Can it be expected of me to share those things as part of my job?” (FGI 3).* For some ESP, it was difficult to distinguish if there was a difference between personal and professional values and if this affected the degree of exposure. The ESP described that healthcare personnel have the need to deliberate on ethical values in a separate forum of their own where they do not risk saying something that might harm or burden families. The need for a separate forum was considered particularly important as it was perceived that healthcare personnel would not have the right to refrain from CESS with PPP if it were to be included in their job description.

## Discussion

This study explored Nordic ESP’s perceptions of PPP in CESS in pediatric oncology. Our results indicate that ESP perceived that PPP in CESS could potentially deepen the common understanding and trust and catalyze confrontation between participants, as well as create dilemmas of decision-making participation. The ESP expressed arguments for potential benefits of PPP in CESS. However, these were overshadowed by potential negative consequences which also permeated the suggested strategies to avoid the risk of causing harm. It appeared to be difficult to conclude with a universal form for, and moral viewpoints on PPP in CESS in pediatric oncology. It was important to customize PPP in CESS and assess whether it was appropriate on a case-by-case basis.

A starting point for discussing PPP in CESS seemed to be the vulnerability of participants, and especially if stakeholders participated together. The vulnerability aspect was a particularly interesting finding as it may suggest that there are some relevant differences between conversations held during CESS compared to other healthcare situations. Indeed, CESS dialogue has a focus on values, is structured, can involve several participants and is led by an (unfamiliar) ESP [5]. Except for this, it ought to involve the same ingredients as other day-to-day ethics conversations between patients and healthcare personnel [36]. The ESP elaborated on potential dilemmas of decision-making participation created by PPP in CESS, and questioned if concerns regarding, for example, uncertainty of how to proceed with a certain medical treatment was ethically defensible to face families with. The ESP advocated this situation as an especially difficult discussion they wanted to spare families. This concern is worthy of respect, but to withhold patients and parents from CESS due to perceived vulnerability (regarding how to handle information for instances) might result in a somewhat unfavorable paternalistic view [37, p. 232]. The perception of the ESP that families cannot handle certain information in CESS can be seen as an example of disguised paternalism [38] as the healthcare personnel’s own values of PPP in CESS unintentionally affect how the patient is treated. Even though hard paternalism (when the patient is competent) has decreased in Nordic healthcare context according to Lynøe and colleagues [38], a soft paternalistic approach (when the patient is not competent) to participation is defensible in many respects, as most of the medical decisions are rightly made by the physicians - thus leading to a limited decision-span for patients and parents. Lynøe et al. [39] describe that healthcare personnel’s personal values can hinder what kind of information a patient receives, thus limiting the shared-decision process that CESS might involve and the patient centered care. As in this study, participation is often discussed in connection to decision-making [14, 40, 41], and it may be of value to reflect upon whether this soft paternalistic view ought to be translated into the ethical decision-making process that CESS might involve. Moreover, as the Convention on the Rights of the Child is incorporated into Swedish law [42], children’s participation in CESS can enable healthcare to fulfill Article 12 which implies the right of children to express their views and to be heard, in accordance with their age and maturity, in all situations that affect them. Nevertheless, the goal of CESS is not always decision-making, rather reflecting on an ethical dilemma in a shared learning process [5].

It is easy to sympathize with the ESP’s fear of causing participant harm, but at the same time it is important to question if the fear is well-grounded. Indeed, it was shown in a Norwegian study [20] that participating in CESS as a next of kin was stressful psychologically, nevertheless no one regretted it, and they experienced it as valuable to hear and express perspectives. It has also been shown that PPP in CESS could reduce tensions between healthcare personnel and patients, and that patients’ direct participation also can clarify the content of the ethical dilemma [2]. Further, the ESP’s fear of causing harm should also be weighed against the potential harm of not involving patients and parents in the structured ethics dialogue. An example showing how the parental view can change the way of action, is from a Norwegian study in neonatal healthcare [43] where pediatricians were asked whether or not to give life-supporting treatment if the extreme preterm patient was their own child. The results showed there was a vital difference between how they would have acted as physician versus parent.

In this study, the results mainly focus on how to manage potential negative consequences, as these were perceived as especially important to implement/improve PPP in CESS. These empirical findings stimulate the normative question of whether we should strive for PPP in CESS or not. Since PPP in CESS seemed like an ethical dilemma in itself with conflicting values involving moral gains and losses, it could be important to highlight the moral reasons for PPP. If Nordic pediatric healthcare finds PPP in CESS normative defensible, they should strive to conduct and improve it despite the ethical challenges, as with the procedure of collecting informed consent [44, 45]. Informing patients about risks can be stressful and potentially harmful for them, but this is not a moral or legal reason to not do so.

This study contributes to a deepened understanding of the perceived complexity of performing PPP in CESS in pediatric oncology and strategies on how to handle the difficulties that may arise. Similar conditions and strategies to facilitate child participation have also been found in social well fare work [46]. The implementation of CESS has been described as a “complex intervention” [47], and certainly our results suggest that PPP in CESS in pediatric oncology may also fall under that concept. Previous research of enablers and barriers of CESS in pediatric oncology [6] also indicate that organizational aspects like the timing of CESS can be of importance. It is important to bear in mind that PPP in CESS in pediatric oncology is in its infancy in research and experience, thus we need to explore, develop and examine it before we make any empirical establishment of its being or not being. The findings in this study need to be further explored and validated.

### Clinical implications

The perceived strategies to avoid causing harm are presented on different levels and we suggest that the organizational conditions are the foremost important aspects to start with. This involves primarily workplace cultural aspects like challenging the ambiguity of ethics and CESS, and to give the ESP relevant and enough education, experience and confidence to become secure facilitators of CESS to introduce PPP. In addition, this study may help educators of CESS in pediatric oncology to understand what the ESP need to implement and improve PPP in CESS. The results may also serve to assist other pediatric clinical settings that are interested in exploring PPP in CESS.

### Strengths and limitations

Most ESP had no former experience of PPP in CESS which means the results mirror hypothetical reasoning, often based on the ESP’s general clinical experience as healthcare personnel and/or as ESP in pediatric oncology (and/or in general pediatrics). This can also affect how the proposed strategies to avoid the risk of causing harm may serve its purpose. It is important to acknowledge that most ESP were educated as facilitators of moral case deliberations, and that their attitudes regarding PPP in CESS therefore may reflect moral case deliberation specifically, and no other forms of CESS. On the basis that a majority of the ESP spent less than one hour per week performing CESS, one can imagine that the organizational challenges described during the implementation of moral case deliberations [31] are still existing and might hinder improvement of PPP in CESS. Thus, discussions about introducing PPP in CESS could be overwhelming and might affect attitudes and perceptions as the ESP themselves probably would be the ones responsible for the PPP implementation. Most ESP had some relation to all authors, either through work or educational contexts, which might have affected their answers. However, the authors’ engagement in pediatric oncology and/or CESS education are seen as strengths as it gives the opportunity to take actions to improve CESS based on the results.

## Conclusions

This study indicates that ESP in pediatric oncology can see arguments for PPP in CESS. However, it is clear that the fear of causing harm overshadows the benefits, which can explain why it has not previously been implemented. The appropriateness of PPP in CESS in pediatric oncology depends on the situation and in cases where it can be considered it does not seem to be any universal way of doing it, but to customize it on a case-by-case basis with focus on reducing the risk of causing participant harm. As PPP in CESS in pediatric oncology sometimes can also be morally challenging and given the limited experience and research in this area, more explicit moral reasoning and practical experiences are needed to foster it in a feasible and morally appropriate way. It will be helpful to highlight the normative arguments for and against PPP in CESS in pediatric oncology more clearly. In addition, ESP’s collected concerns, both practically and morally, are insightful and can help us in starting well prepared pilots of PPP in CESS in pediatric oncology, combined with participatory evaluation research. In this way, this study will contribute to foster the potential advantages of a practically and morally appropriate PPP in CESS in pediatric oncology while considering the concerns and potential harm of it.

## Electronic supplementary material

Below is the link to the electronic supplementary material.

Supplementary Material 1

## Data Availability

If ethical approval is obtained, data is available on reasonable request. The interview guide is available as additional file.

## Abbreviations

CESS: Clinical ethics support services
PPP: Patient and parent participation
ESP: Ethics support personnel
FGI: Focus group interview
