# Supplementary material for: Ethics support personnel’s perceptions of patient and parent participation in clinical ethics support services in pediatric oncology
Source: BMC Med Ethics. 2025 Jul 19;26:104. doi: 10.1186/s12910-025-01267-5 (PMC12275289; doi:10.1186/s12910-025-01267-5)
Supplement: Supplementary file 1 — Supplementary Material 1 [file 12910_2025_1267_MOESM1_ESM.pdf]

Karolinska Institutet

Interview guide – Healthcare professionals/other staff with experience of clinical ethics support services (CESS)

We have now talked about how healthcare professionals, guardians and children can get help to handle ethical dilemmas. Now we would like to hear your thoughts about this.

- What do you know about CESS that are available at your clinic?
  - How could children and guardian participate in those activities?
- How does your clinic offer children and guardians CESS today?
- In general, what are your thoughts of children and guardian participation in CESS?
  - What advantages are there with their participation?
  - What risks are there with their participation?
- What enables and prevents you from performing CESS with children and guardian participation?
  - Personal promoting/inhibiting factors
  - Organization/practical promoting/inhibiting factors

Case 1. *A family must decide whether their child, newly diagnosed with cancer, will participate in a research study including randomization to different treatments. The guardians are very uncertain about what is the right thing to do and are afraid that the child will receive inadequate treatment or unnecessary side effects.*

If you were the healthcare /ethics support professional in a similar situation:

- What are your thoughts of how you/your clinic could offer CESS with patient and guardian participation in this situation?
  - Who would participate (in the activities)?
  - What would be the goal of the activity?
  - What disadvantages are there and what inhibit the different activities?
  - What advantages are there and what enables the different activities?

Case 2. *Standard treatment is not working and there is a great medical uncertainty among stakeholders which treatment to proceed with. Decisions need to be made on other grounds than purely medical ones (values) and there are major doubts of what is best/right.*

If you were the healthcare/ethics support professional in a similar situation:

- What are your thoughts of how you/your clinic could offer CESS with patient and guardian participation in this situation?
  - Who would participate (in the activities)?
  - What would be the goal of the activity?
  - What disadvantages are there and what inhibit the different activities?
  - What advantages are there and what enables the different activities?

Case 3. *The teenager wants to receive care at home, but the parent/s does/do not want to leave the hospital because of fear.*

If you were the healthcare/ethics support professional in a similar situation:

- What are your thoughts of how you/your clinic could offer CESS with patient and guardian participation in this situation?
  - Who would participate (in the activities)?
  - What would be the goal of the activity?
  - What disadvantages are there and what inhibit the different activities?
  - What advantages are there and what enables the different activities?

Case 4. *The parents disagree with each other about what is the best for the child.*

If you were the healthcare/ethics support professional in a similar situation:

- What are your thoughts of how you/your clinic could offer CESS with patient and guardian participation in this situation?
  - Who would participate (in the activities)?
  - What would be the goal of the activity?
  - What disadvantages are there and what inhibit the different activities?
  - What advantages are there and what enables the different activities?

Case 5. *The parents do not want the healthcare professionals to tell the child that curative treatment has failed. The healthcare professionals perceive that the child wants to know but does not dear to ask.*

If you were the healthcare/ethics support professional in a similar situation:

- What are your thoughts of how you/your clinic could offer CESS with patient and guardian participation in this situation?
  - Who would participate (in the activities)?
  - What would be the goal of the activity?
  - What disadvantages are there and what inhibit the different activities?
  - What advantages are there and what enables the different activities?

- Do you have more thoughts on how patient and guardians can participate in CESS?

Potential follow up question to all cases:

- Can you tell us more?
- Can you give an example?
- How would that make you feel? Why do you think it would make you feel like that?
- How come you think like that?
- What would happen then?
